# Supplementary material for: Free Cholesterol Affects the Function and Localization of Human Na+/Taurocholate Cotransporting Polypeptide (NTCP) and Organic Cation Transporter 1 (OCT1)
Source: Int J Mol Sci. 2022 Jul 30;23(15):8457. doi: 10.3390/ijms23158457 (PMC9368832; doi:10.3390/ijms23158457)
Supplement: Supplementary file 1 [file ijms-23-08457-s001.zip › ijms-1823562-supplementary.pdf]

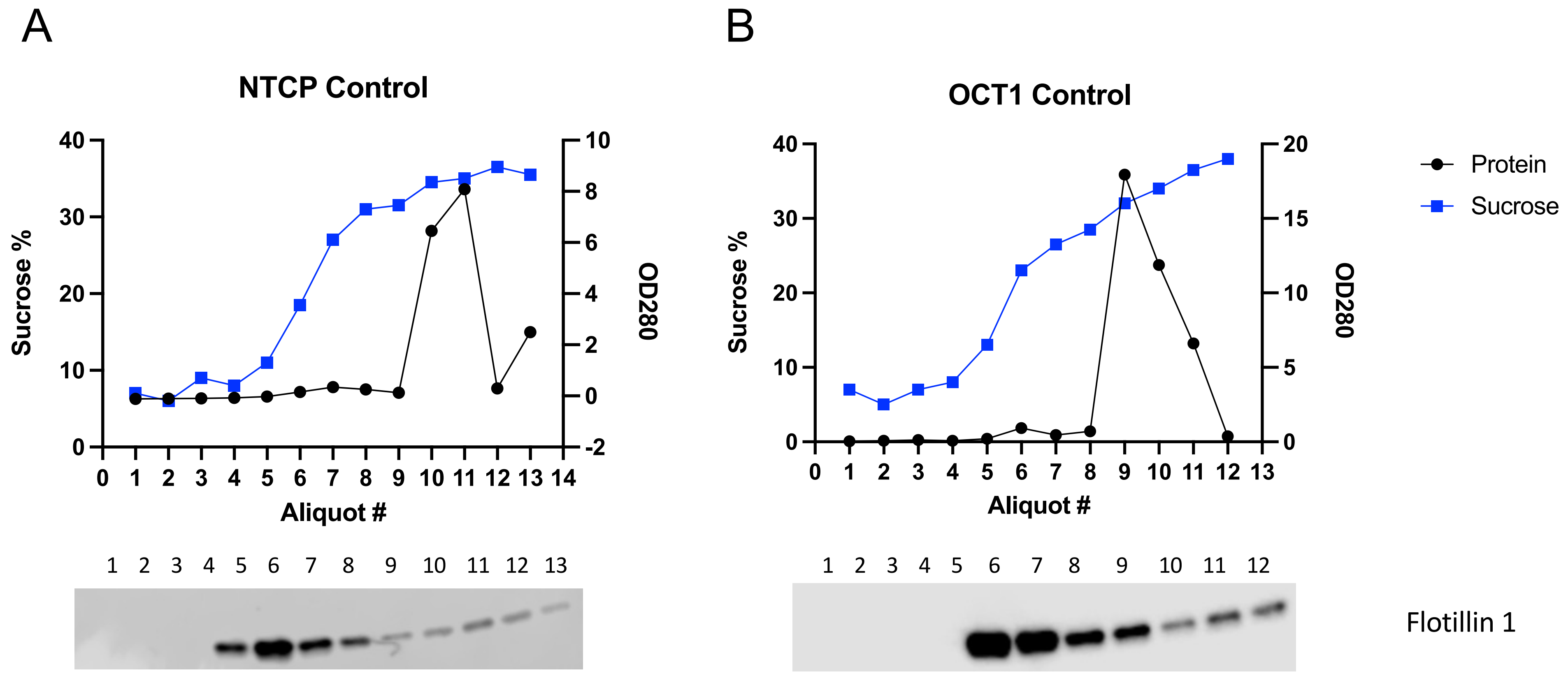

Figure S1: Distribution of total protein, sucrose concentration, and the lipid raft marker protein flotillin 1. After homogenization and incubation with TX-100, samples were mixed with a sucrose solution to yield 40% and overlayed with 35 and 5 % sucrose, respectively (see Materials and Methods for more details). After an 18-hour centrifugation, 1ml fractions were collected, sucrose concentration was determined with a refractometer, and protein concentration was measured using OD280. Samples were analyzed for the lipid raft marker Flotillin 1 by western blotting.
